# Rare Fibroepithelial Polyp Extending Along the Ureter: A Case Report

**DOI:** 10.4274/balkanmedj.2017.1537

**Published:** 2018-05-29

**Authors:** Hakan Akdere, Gökhan Çevik

**Affiliations:** 1 Department of Urology, Trakya University School of Medicine, Edirne, Turkey

**Keywords:** Children, fibroepithelial, polyps, urinary tract

## Abstract

**Background:**

Fibroepithelial polyps of the urinary tract are rare tumors, and their occurrence in the upper urinary tract is highly unusual.

**Case Report:**

This study reports a 9-year-old boy who presented to our clinic with complaints of unilateral flank pain and macroscopic hematuria. The direct urinary system graph did not show stone formation; therefore, magnetic resonance urography was performed. This revealed a filling defect in the left proximal ureter. On cystoscopy, a polyp was seen in the orifice of the left ureter, extending along the ureter. The polyp was resected by laser ablation and removed from the ureter. Histopathologic examination revealed a fibroepithelial polyp comprising fibrovascular stroma covered with transitional epithelium.

**Conclusion:**

Although extremely rare, a fibroepithelial polyp should be considered in the differential diagnosis when a young patient presents with flank pain and macroscopic hematuria. Endoscopic procedures may be the treatment of choice for polyps located in the upper ureter.

Fibroepithelial polyps (FEPs) are rare, benign, non-epithelial tumors of mesodermal origin that are located in the urinary system. They originate in the stroma, which consists of mesodermal and normal transitional epithelial cells. Typically, FEPs are found in the upper urinary system of adolescent patients. The mean diameter of all reported FEPs is less than 5 cm, and they occur as plain, mobile, and pedunculated masses (1). Although most FEPs are encountered in the distal ureter, 15% are located in the renal pelvis; FEPs are found less often in the urethra, bladder, and proximal ureter (1). They are usually unilateral, with the left ureter involved twice as often as the right ureter (2). FEPs should be considered after a radiolucent filling defect in intravenous pyelography (IVP) or retrograde ureterography and negative cytology (3). Because FEPs are usually located in the distal ureter, most can be diagnosed and followed up in the same way as for a congenital ureter stenosis. FEPs can cause hydronephrosis in adults (4). This study reports a case of an FEP that originated in the ureteropelvic junction (UPJ) and extended along the ureter. Polyps with these features are rare among children. The polyp was successfully treated with endoscopic laser ablation.

## CASE PRESENTATION

**Figure 1:**
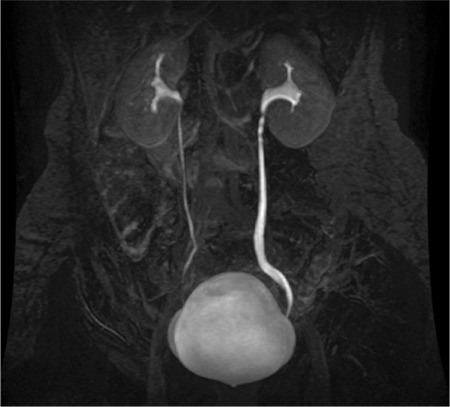
The filling defect in the left proximal ureteropelvic junction on magnetic resonance urography.

**Figure 2:**
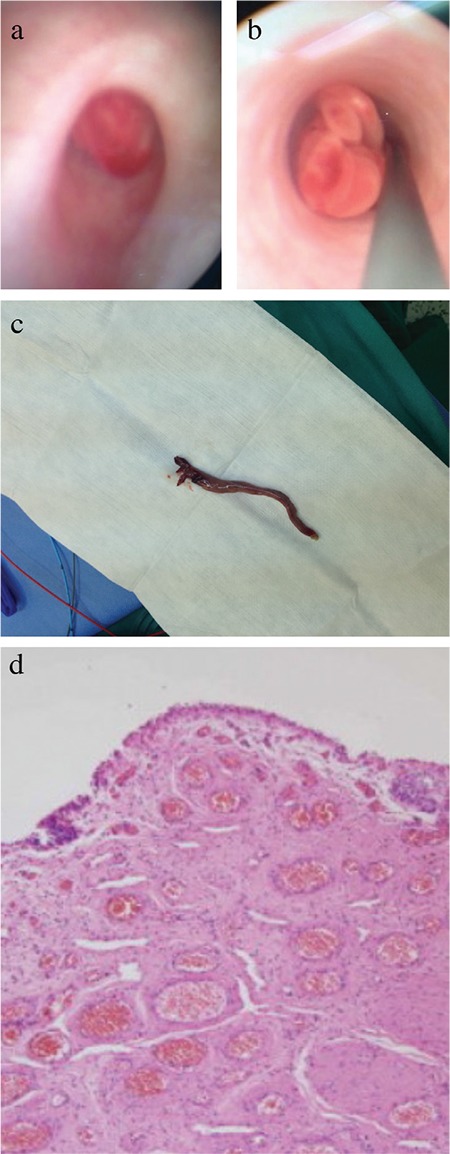
The polyp from the orifice (a), the polyp in the ureter (b), the resected polyp (10 cm) (c), transitional epithelium covering the fibrovascular stroma (d) (H&E, x100).

A 9-year-old boy presented to our clinic with fever, nausea, vomiting, and colic pain on his left side. Physical examination revealed sensitivity in the costovertebral angle and ureteral points. Urinalysis indicated macroscopic hematuria. The results of the biochemical examination were normal; however, ultrasonography (USG) revealed grade 2 dilatation in the pelvicalyceal system of the left kidney. The patient was suspected with ureteric stone, and a direct urinary system graph was obtained during pain control with analgesia. However, this did not reveal any stone formation. Therefore, magnetic resonance urography was performed; this revealed a filling defect in the left proximal ureter (Figure 1), which was clinically and radiologically considered to be an urothelial carcinoma. Cystoureteroscopy was performed. A pedunculated polyp from the orifice to the UPJ was detected, and the stalk of the polyp was separated from the ureter wall using a Ho: YAG laser (Figure 2a, b). Subsequently, the entire tissue (10 cm) was removed with forceps (Figure 2c). There were no postoperative complications, and the patient was discharged on the first postoperative day. Laboratory tests performed during the first postoperative week were normal. USG revealed no sign of ectasia in the left kidney. Histopathologic examination of the polyp under a microscope revealed edema covered by fibrovascular stromal tissue and a fibroepithelial structure composed of normal transitional epithelium with distinctive vascularization (Figure 2d). Written informed consent was obtained from the patient's parents.

## DISCUSSION

FEPs are rare tumors and are generally located in the distal ureter. Their occurrence in the UPJ is highly unusual (5). The treatment method for children should be considered very carefully because of the potential for postoperative complications, including the development of a stricture that may lead to renal failure (6).

The etiology of benign ureteral polyps is uncertain. They are thought to be a congenital abnormality acquired or caused by an infection, chronic irritation, obstruction, or trauma (3). Although congenital causes are more common in children, most FEPs result from inflammation or infections in adults. FEPs can be involved in stone formation and obstructions, and they can be confused with malignant tumors (5). The most common presenting symptom of FEPs is hematuria accompanied by side pain. FEPs usually appear as long and straight ureteral filling defects in IVP and retrograde ureterography with or without hydronephrosis. However, FEPs cannot be diagnosed with imaging alone. On ureteroscopy, the smooth and regular appearance of FEPs can easily be differentiated from irregular urothelial carcinomas (5). Therefore, a biopsy should be performed in all cases to obtain histologic proof before starting the definitive therapy (1). Treatment for these polyps is determined by the degree of obstruction, the existence of a urinary tract infection, and the intraoperative suspicion of malignancy (2). Some authors have reported that favorable results could be obtained using the percutaneous and/or ureteroscopic approach, particularly when assisted by endoscopic and laparoscopic devices (6). Lam et al. (1) reported five patients with FEP; three were treated with percutaneous electroresection and two by ureteroscopy with Ho: YAG laser ablation. Carey and Bird (5) used a flexible ureteroscope to detect the basis of a polyp on the ureteral wall, and they treated 10 patients with ureteroscopic laser ablation. Kijvikai et al. (7) reported a case with a 17-cm-long FEP stemming from a distal UPJ, which was treated with laparoscopic pyeloplasty. Recently, endoscopic treatment has been used with increasing frequency for patients with ureteral tumors, as seen in the present case (8). Open or laparoscopic/robotic treatment options may be available for surgeons who lack sufficient experience for endoscopic treatment (9). Several features made this case unique: the localization of the polyp extending along the ureter; the treatment; and the successful endoscopic treatment without postoperative stricture. Although extremely rare, FEP should be considered in the differential diagnosis when a young patient presents with flank pain and macroscopic hematuria.
